# Topographic Reorganization of EEG Complexity During Visual Mental Imagery: Insights from Lempel-Ziv Complexity in High-Density EEG

**DOI:** 10.1007/s10548-026-01238-y

**Published:** 2026-07-21

**Authors:** Yu Gao, José Miguel Diniz

**Affiliations:** 1 Department of Electrical and Computer Engineering, Faculty of Engineering, University of Porto, Porto, Portugal; 2 CISTER/ISEP - Research Centre in Real-Time and Embedded Computing Systems, School of Engineering, Porto, Portugal; 3 PhD Program in Health Data Science, Faculty of Medicine, University of Porto, Porto, Portugal; 4 Faculdade de Engenharia da Universidade do Porto, Rua Dr. Roberto Frias, 4200 – 465 Porto, Portugal

**Keywords:** EEG, Mental Imagery, Neural Complexity, Lempel-Ziv Complexity, Higuchi Fractal Dimension, Cortical State Decoding

## Abstract

Visual mental imagery is the process of reconstructing perceptual experience without sensory input. How the brain performs this process is poorly understood, particularly from the perspective of conventional linear EEG analysis. This study aims to evaluate if the two non-linear EEG complexity measures—Lempel-Ziv Complexity (LZC) and Higuchi Fractal Dimension (HFD)—can differentiate between perception and imagination and if they can be used as objective indices of neural separability of mental imagery. LZC and HFD were extracted from 62 scalp EEG channels in 46 healthy adults performing the PerceiveImagine paradigm (Li and Fan 2024), after wideband Picard ICA decomposition (1–200 Hz), which was used to make residual artefacts explicit rather than to remove components, with edge-channel EMG monitoring for artefact control. Statistical analyses included cluster-based permutation testing (Maris and Oostenveld 2007), Hotelling T², and leave-one-subject-out cross-validation (LOSO-CV). Broadband LZC topography differed between perception and imagination (cluster *p* = 0.005; Hotelling F = 3.08, *p* = 0.002, V = 0.28). LOSO-CV classification reached AUC = 0.811 (95% CI: [0.775, 0.847]). The classifier’s AUC exceeded a label-permuted baseline by a wide margin (t(45) = 16.30, *p* < 0.001). The a priori low-gamma LZC hypothesis was not supported, with no significant difference at the occipital ROI (*p* = 1.0, d = − 0.13). At the scalp level, EEG complexity features are associated with a topographic redistribution rather than a global magnitude change: imagery shows a relative posterior-to-frontal shift in broadband LZC. Because these patterns are scalp-recorded, they characterise the spatial distribution of complexity rather than establishing the underlying cortical sources or the direction of information flow. Objective decoding-confidence labels provide a more usable training signal than the (invariant) self-report available in this dataset, indicating future potential for imagery-quality indexing rather than immediate translational readiness.

**Supplementary Information:**

The online version contains supplementary material available at https://doi.org/10.1007/s10548-026-01238-y.

## Introduction

Visual mental imagery refers to the ability to internally reconstruct perceptual experience without external sensory input. When a person imagines an apple, primary visual cortex activates in proportion to the vividness of that image, despite the absence of any retinal signal (Dijkstra et al. 2018). Understanding how the brain accomplishes this reconstruction has practical relevance for brain–computer interfaces, neurofeedback, and cognitive rehabilitation. Most EEG studies still focus on linear spectral qualities, such as band power, which likely overlooks the irregularity of the endogenously determined neural processes.

Two non-linear metrics, however, provide a different perspective. Lempel-Ziv Complexity (LZC) measures the number of distinct sequential patterns in a binarized signal: the more patterns, the greater the signal’s temporal irregularity. Higuchi Fractal Dimension (HFD) determines the scaling of a time series’ curve length across various time resolutions, producing a Fractal Dimension that indicates geometric roughness. The two indices are intended to capture complementary, rather than strictly orthogonal, facets of neural dynamics: LZC reflects the symbolic novelty of the median-binarized signal, whereas HFD quantifies the geometric scaling (fractal roughness) of the continuous waveform across time resolutions. Both are nonetheless known to be influenced by spectral content and signal non-stationarity, so their degree of independence is an empirical question rather than an assumption; we therefore quantify their cross-channel relationship directly (Sect.  Relationship Between LZC and HFD, and Incremental Value) rather than presupposing redundancy or complementarity. Used jointly, they provide a richer characterisation of non-stationary EEG dynamics than either index alone.

LZC and HFD sit within a broader family of non-linear EEG descriptors that have been widely applied in neuropsychology and neuropsychiatry. Entropy-based measures such as Approximate Entropy (ApEn), Sample Entropy (SampEn) and Permutation Entropy (PermEn) quantify the regularity or predictability of a time series, while methods such as Singular Spectrum Analysis (SSA) and autoregressive (AR) model order characterise its underlying dynamical structure; these metrics have demonstrated utility in indexing altered complexity in aging, internal mentation and a range of neurological and psychiatric dysfunctions (Aydın 2010; Aydın et al. 2011; Aydın and Akın 2022; Burns et al. 2015). We selected LZC and HFD for three reasons. First, both are computationally lightweight and deterministic, avoiding the embedding-dimension and tolerance-parameter sensitivities that complicate ApEn/SampEn estimation on short epochs. Second, they probe conceptually distinct properties—symbolic sequence diversity (LZC) and geometric self-similarity (HFD)—rather than two variants of the same regularity statistic. Third, both have recently been validated as markers of cortical state and region-specific dynamics in scalp EEG (see below). We do not claim these two indices are superior to entropy- or model-based alternatives; a systematic comparison against ApEn, SampEn, PermEn and spectral/connectivity features is an explicit goal for future work (Sect.  Classification and the Objective Label Advantage).

Recent studies have confirmed that the two metrics are responsive to the different brain states. Höhn et al. (2024) showed that broadband LZC and spectral slope together monitor sleep/wake states in healthy adults. Medel et al. (2023) found that LZC and the 1/f spectral slope provide a complementary description of the excitation-inhibition balance in the cortex. Ren et al. (2023) applied LZC to resting state EEG, and were able to identify cognitive impairment in epilepsy patients. On the HFD side, Aggarwal and Ray (2025) reported that HFD in scalp EEG is anticorrelated with oscillatory power and 1/f slope across the adult lifespan. Colussi et al. (2025) used HFD to differentiate wakefulness from sleep in a developmental cohort. Armonaite et al. (2023) validated HFD as a region-specific cortical complexity signature, and Ruiz de Miras et al. (2023) applied fractal dimension analysis to resting-state EEG networks in schizophrenia. Despite this growing body of work, no study has jointly examined LZC and HFD as complexity descriptors of visual mental imagery decodability.

The imagery literature itself has an unresolved measurement problem. Dijkstra et al. (2021) found that perception and imagery share neural substrates in visual cortex but differ in temporal dynamics—imagery recruits top-down feedback more slowly and with greater trial-to-trial variability. Chang et al. (2025) released a multisensory EEG imagery dataset with vividness ratings, confirming that subjective vividness correlates with distinguishable EEG power patterns. Wilson et al. (2024) reviewed the feasibility of visual decoding from EEG. Shimizu and Srinivasan (2022) improved classification of imagined images using spectral features. Gifford et al. (2022) provided a large benchmark EEG dataset for visual object recognition, and Lee et al. (2022) explored the classification of perception versus imagery. The common thread across these studies is a reliance on subjective vividness ratings as the only ground truth for imagery decodability, even though self-report is noisy and inconsistent across sessions. An objective approach is to classify perceptual and imagined states, and use the classifier’s decoding confidence as a continuous quality measure.

We incorporated this dual-labeling method with our broadband artefact-control processing pipeline. The gamma-band range (30–150 Hz) overlaps with scalp muscle artefact, which, if unchecked, can confound complexity differences. Instead of removing the high frequencies, we applied Picard ICA (Ablin et al. 2018) over the entire 1–200 Hz range; and used EMG power monitoring as a secondary protective measure.

We evaluated eight hypotheses related to 46 participants in the PerceiveImagine paradigm (Li and Fan 2024) and extracted 620 complexity features per trial (434 LZC + 186 HFD) from 62 scalp EEG channels. The gamma-band LZC elevation we predicted did not occur. Instead, we observed a significant topographic redistribution of broadband complexity between perception and imagination. This finding is in line with predictive coding frameworks suggesting imagery redistributes the cortical dynamics, rather than elevating their magnitude uniformly.

**This study makes three contributions.** Firstly, it shows that broadband EEG complexity, measured at the scalp, distinguishes perception from imagery through a spatial reallocation of complexity rather than a uniform magnitude change—a pattern that is consistent with, but does not by itself establish, the reversal of cortical information flow posited by top-down generative models. Secondly, it highlights that decoding-derived confidence surpasses chance-level baselines with strong statistical evidence (t(45) = 16.30, *p* < 0.001), thus qualifying it as a valid objective measure of imagery decodability. Third, it validates a reproducible pipeline for shuffle-normalized LZC and sliding-window HFD extraction on a 46-subject public dataset. Code is publicly available at https://github.com/jabarrick/LZC-HFD-hypothesis.

We hypothesized that mental imagery would result in varying complexity signatures in the low-gamma band (30–60 Hz). This a priori choice is based on theory regarding the role of gamma-band neural synchrony in endogenous neural binding and top-down sensory reconstruction. By isolating this band and employing a rigorous wideband ICA artifact-control pipeline, we aimed to determine whether reported gamma-band complexity changes reflect genuine neural dynamics or are susceptible to scalp muscle contamination.

## Materials and Methods

### Dataset and Participants

We analyzed the PerceiveImagine dataset (OpenNeuro ds005697) (Li and Fan 2024), which was collected from 54 healthy adults at a university laboratory in China. Eight participants were not included in the present analysis: seven had missing or corrupted data files (sub-07, sub-12, sub-13, sub-39, sub-51, sub-52, sub-53, as documented in the dataset release notes), and one (sub-38) was excluded because its behavioural log contained no perception or imagination event codes, so that zero valid epochs could be extracted. The final analytic sample consisted of 46 participants, aged 23 to 30, both male and female, with no reported neurological or psychiatric history and normal or corrected-to-normal vision.

### Data Acquisition

**Fig. 1:**
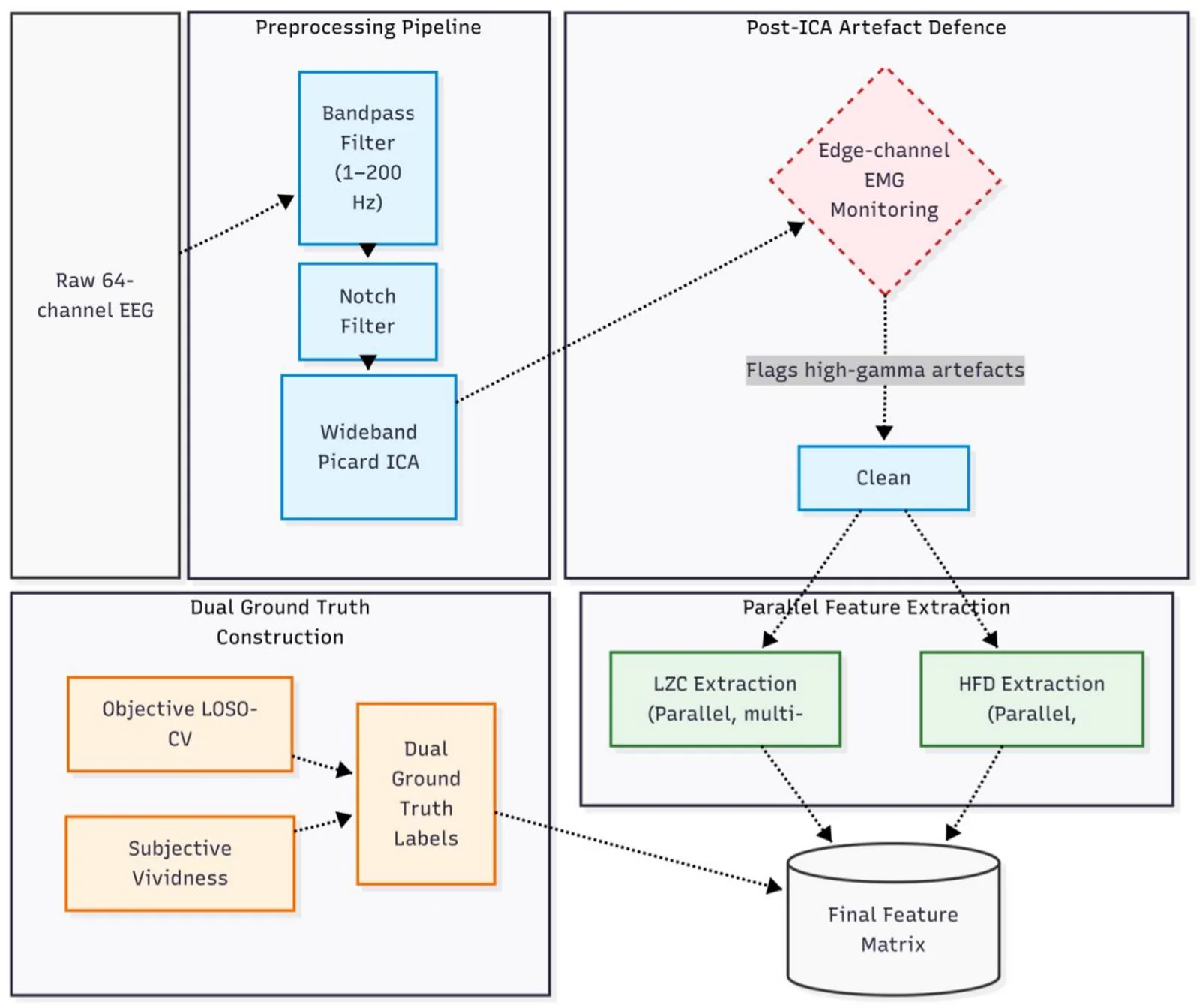
End-to-end preprocessing and feature extraction pipeline. Raw 62-channel EEG is bandpass-filtered (1–200 Hz), notch-filtered, and processed with wideband Picard ICA decomposition. Post-ICA edge-channel EMG monitoring flags high-gamma artefacts. Clean epochs undergo parallel LZC and HFD extraction. Dual ground truth labels are constructed from LOSO-CV decoding confidence

EEG was recorded at 1000 Hz with a Neuroscan SynAmps2 system in a standard 10–20 electrode layout. The recording montage comprised 62 EEG channels for analysis, corresponding to a 64-channel 10–20 layout without the AF7 and AF8 sites and including the two mastoid electrodes (M1, M2); a common average reference (CAR) was computed across these 62 channels. Each experimental trial comprised a fixation cross (2 s), a visual stimulus presentation (6 s), a brief gap (0.5 s), a mental imagination period (6 s), and a vividness self-report. The stimulus set contained 340 naturalistic images. The end-to-end preprocessing and feature extraction pipeline is illustrated in Fig. 1.

### Preprocessing

All preprocessing was carried out in MNE-Python (≥ 1.6). The continuous data were bandpass-filtered at 1–200 Hz using a zero-phase FIR filter and notch-filtered at 50, 100, and 150 Hz to remove power-line harmonics. The wideband upper edge of 200 Hz was chosen deliberately. Although classical scalp EEG oscillatory activity is generally considered reliable only up to approximately 100 Hz—beyond which myogenic (EMG) contamination becomes increasingly prevalent—our a priori hypotheses concerned gamma-band complexity, the very range in which prior reports are most vulnerable to muscle artefact. Rather than band-limiting the data and thereby pre-empting the question, we retained the full range and addressed contamination explicitly through wideband ICA and edge-channel EMG monitoring (below), so that any gamma-band effect could be evaluated under stringent artefact control rather than assumed absent or genuine. We acknowledge the trade-off: wideband processing increases exposure to residual EMG, and we therefore interpret high-gamma results conservatively. A reduced-bandwidth sensitivity analysis (< 100 Hz low-pass) is planned as future work (Supplementary Table S1); the 200 Hz upper limit used here is conservative for cortical EEG and consistent with prior high-density EEG complexity studies. A common average reference was applied. We then fitted Picard ICA (Ablin et al. 2018) on the wideband signal with extended decorrelation enabled (random seed = 42; number of components set to the data rank). The pipeline was configured to classify components automatically with ICLabel and to remove those labelled “muscle” (probability > 0.6) or “eye”. However, reliable automatic ICLabel classification could not be obtained for this dataset, which lacks individual electrode digitisation; consequently no independent components were removed, and the ICA decomposition served to make any residual ocular or myogenic contribution explicit rather than to subtract it. Artefact control therefore rested on the wideband-decomposition design together with the edge-channel high-gamma EMG monitoring described below, and we interpret high-gamma results conservatively in light of this limitation.

As a further layer of artefact control, we computed the mean high-gamma band power (80–150 Hz) at six edge channels (T7, T8, TP7, TP8, PO7, PO8) for each imagination epoch via Welch periodogram. Any epoch whose edge-channel power exceeded 3.0 standard deviations above the per-subject mean was flagged, following standard artefact rejection practice. In a verification subset of five subjects, approximately 1.2% of imagination epochs were flagged (mean 1.18%, range 0.3–2.1%), consistent with a low residual-EMG burden. Flagged epochs were removed only from high-gamma analyses; broadband and sub-gamma computations retained them.

### Epoching

Event information was parsed exclusively from the behavioural log files (events.tsv) rather than from hardware trigger channels, because some recordings contained spurious trigger pulses not present in the behavioural logs. A three-tier cascading classifier assigned event labels to conditions: keyword matching was tried first, then numeric prefix rules (labels starting with “1” mapped to perception, “2” to imagination), and finally an ordinal fallback for any remaining unmatched labels. Epochs were extracted with a − 0.2 to 0.0 s baseline and a post-onset duration of 6.0 s. The resulting master feature table contained 30,534 trials across all 46 subjects, with approximately 331 perception trials and 332 imagination trials per subject, corresponding to the 340-image stimulus set minus occasional parsing losses.

### Lempel-Ziv Complexity

LZC measures a signal’s irregularity in time by evaluating the number of unique sub-patterns formed in a sequential left-to-right scan of a binary sequence. This process involves five steps.

**Step 1 – Bandpass filtering.** The continuous signal was filtered into six sub-bands using a fourth-order Butterworth filter delta: 1–4 Hz, theta: 4–8 Hz, alpha: 8–13 Hz, beta: 13–30 Hz, low-gamma: 30–60 Hz, high-gamma: 60–150 Hz). Along with the unfiltered broadband signal (1–200 Hz), this provides a total of seven frequency conditions.

**Step 2 – Median binarization.** The filtered signal x(n) of length N was converted into a binary sequence using the median as the threshold, which is a standard convention in LZC computation and is robust to non-Gaussian amplitude distributions:

1 $$ s\left( n \right){\text{ }} = {\text{ }}1{\text{ }}if{\text{ }}x\left( n \right){\text{ }} \ge {\text{ }}median\left( x \right),~~0{\text{ }}otherwise $$

**Step 3 – LZ76 parsing.** We analyzed the binary sequence using the Kaspar–Schuster variant of the LZ76 algorithm, which, via a pointer-increment scheme, counts the number of novel substrings c(n). We compiled our implementation to native machine code using Numba’s @njit decorator, which also validates import—an all-zeros sequence produces c = 1 and an alternating 0–1 sequence produces c = 2.

**Step 4 – Length normalization.** The raw complexity count was normalized against the theoretical upper bound for a random binary string of length N:

2 $$ LZC\_norm{\text{ }} = {\text{ }}c\left( n \right){\text{ }}\cdot{\text{ }}log\left( N \right){\text{ }}/{\text{ }}N~ $$

**Step 5 – Shuffle normalization.** To isolate structural complexity from the marginal statistics of the signal (Medel et al. 2023), 100 surrogate sequences were generated by Fisher-Yates shuffling. The LZC value is computed as the observed LZC_norm divided by the mean LZC_norm of the surrogates. Values near 1.0 suggest complexity comparable to a random sequence, whereas values below 1.0 suggest temporal regularity. The distribution of surrogate-normalised LZC across all subjects is shown in Supplementary Fig. S1, confirming that values lie well below 1.0 throughout.

This pipeline produced 434 features per trial (7 frequency conditions × 62 channels).

The selection of these two non-linear indices is grounded in their mathematical complementarity: while LZC is sensitive to the temporal irregularity of pattern sequences, HFD is sensitive to the self-similar structure of the time series. Together, they form a multi-dimensional feature space that is robust to inter-subject spectral variability.

### Higuchi Fractal Dimension

HFD estimates the fractal dimension of a time series by measuring how its curve length L(k) scales across integer delay parameters k = 1, 2, …, k_max. For each delay k and starting offset m = 1, …, k, the normalized length is:

3 $$ \begin{aligned} L\_m\left( k \right){\text{ }} = & \left[ {\left( {N - 1} \right){\text{ }}/{\text{ }}\left( {floor\left( {\left( {N - m} \right)/k} \right){\text{ }} \cdot {\text{ }}k} \right)} \right]{\text{ }} \cdot \\& \Sigma {\mid }x\left( {m + jk} \right){\text{ }} - {\text{ }}x\left( {m + \left( {j - 1} \right)k} \right){\mid } \\ \end{aligned} $$

The average over all offsets gives L(k), and HFD is obtained as the slope of log L(k) versus log(1/k) through linear regression. We used vectorized NumPy polyfit to compute the regression across all 62 channels simultaneously. The parameter k_max was set to 64, following values used in previous EEG studies (Aggarwal and Ray 2025; Colussi et al. 2025; Höhn et al. 2024). Olejarczyk et al. (2022) and Armonaite et al. (2023) have shown that HFD functions as a region-specific cortical complexity signature, which supports its use in topographic analyses like the one reported here.

Two temporal resolutions were employed. Epoch-level HFD was computed once over the full 6-second trial (6000 samples at 1000 Hz). Sliding-window HFD was computed in a 1.0-second window advanced in 0.1-second steps, yielding approximately 60 windows per trial. From each channel’s window trajectory, the mean and standard deviation were extracted. The total number of HFD features was 186 per trial (62 channels × 3 statistics: epoch-level, window mean, window SD). To probe transient dynamics and verify that the topographic effect is not an artefact of epoch-length averaging, broadband LZC was additionally computed in sliding 1-second windows (0.1-second steps) across each 6-second epoch and averaged across trials and subjects for whole-scalp, frontal, and occipital ROIs (Supplementary Fig. S7).

### Dual Ground Truth

The PerceiveImagine dataset records a vividness self-report after each trial, but the ratings in this cohort are constant across all subjects and trials (value = 3 on a 1–5 scale). Because there is no within-subject variance in vividness, we adopted a dual ground truth framework instead.

**Objective label.** A LOSO-CV logistic regression classifier (L2 regularization, C = 0.1 chosen to balance bias and variance, L-BFGS solver) was trained to discriminate perception from imagination. The feature vector for each trial comprised the 62 broadband LZC features (one per channel); HFD features were not entered into this classifier and were analysed separately (Sect. Higuchi Fractal Dimension, Relationship Between LZC and HFD, and Incremental Value). To avoid information leakage, all preprocessing of the feature matrix was performed strictly within each training fold: for every left-out subject, the StandardScaler was fitted on the training subjects only and then applied unchanged to the held-out subject, and the logistic-regression coefficients were estimated solely from the training fold. No scaling, feature selection, or model fitting used data from the held-out subject, and no global normalisation was applied before splitting. We did not perform separate dimensionality reduction for the classifier; regularisation (the L2 penalty) was relied upon to control the high feature-to-subject ratio (62 features vs. 46 subjects). For each held-out subject, the predicted probability of the “imagination” class served as a continuous objective quality metric. Per-subject AUC was computed wherever both classes were present. All 46 subjects were valid.

**Proxy subjective label.** Because vividness ratings lacked variance, the mean decoding probability per subject was used as a proxy. Subjects were split at the median into a high-decoding group (*n* = 23) and a low-decoding group (*n* = 23). This design creates a known circularity, which is discussed openly in Sect.  Limitations.

A permutation baseline was also constructed for hypothesis H8: for each subject, the true test-set labels were randomly permuted 50 times, and the resulting mean AUC served as a subject-specific estimate of chance performance (approximately 0.500).

### Statistical Analysis

Eight hypotheses were tested. Cluster-based permutation testing used 1000 permutations with spatial adjacency computed from the 10–20 montage via the MNE built-in channel adjacency matrix (Maris and Oostenveld 2007). This non-parametric procedure controls the family-wise error rate across spatially adjacent channels. The significance threshold was α = 0.05 throughout. The Benjamini–Hochberg procedure was applied to the five hypotheses with conventional, independently interpretable p-values (H1, H2, H3, H4, H8); H5 was assessed against a pre-specified AUC threshold, H7 was untestable, and H6 is a by-construction consistency check (see below), so these three were excluded from the correction.

**H1**: Low-gamma (30–60 Hz) LZC is higher during imagination than perception at six occipital channels (Oz, O1, O2, POz, PO3, PO4). Effect size: paired Cohen’s d.

**H2**: Low-gamma LZC is higher in the high-decoding group than the low-decoding group during imagination. Effect size: independent-samples pooled Cohen’s d.

**H3**: Frontal HFD shows a condition × decoding-quality interaction. Tested via linear mixed-effects model (HFD ~ condition × y_subj_raw + (1|subject)) at five frontal channels (Fp1, Fp2, F3, Fz, F4). Because y_subj_raw is a subject-level variable rather than a trial-level covariate, the effective degrees of freedom for the interaction term are governed by the number of subjects (*N* = 46), not the number of trials.

**H4**: Broadband LZC topography differs between perception and imagination. Tested by both cluster-based permutation and Hotelling T² on PCA-reduced channel vectors (10 components). To maintain a robust observations-to-variables ratio for the multivariate test (N = 46 vs. k = 10), dimensionality was reduced to the first 10 principal components, which accounted for 94.6% of the total topographic variance while capturing the predominant spatial structures. We emphasise that this PCA step applies only to the parametric Hotelling T² confirmation of H4: the primary test of H4 is the mass-univariate cluster-based permutation test, which is computed across all channels without any dimensionality reduction, so the retained-variance fraction bears only on this secondary confirmatory test and not on the primary topographic result.

**H5**: LZC features achieve AUC > 0.72 for state discrimination in the LOSO-CV classifier.

**H6**: High-decoding subjects show higher objective accuracy than low-decoding subjects. Note that the two groups are defined by a median split on the same decoding metric, so this comparison is a by-construction consistency check on the proxy labelling rather than an independent test. Effect size: rank-biserial correlation r_rb.

**H7**: Within-subject Spearman correlation between vividness and decoding accuracy exceeds *r* = 0.3. Not testable due to zero vividness variance.

**H8**: Objective decoding AUC exceeds the permutation-baseline AUC (i.e. real-label decoding outperforms chance). Tested by paired t-test (with Wilcoxon signed-rank as a robustness check). A Bayesian t-test (Cauchy prior, scale *r* = 0.707; Rouder et al. 2009) was also computed but is not reported numerically because the Bayes-factor integral is numerically unstable at the observed large t value.

## Results

### Preprocessing Summary

All 46 subjects passed through the preprocessing pipeline without failure, and Picard ICA converged in every case. As noted in Sect.  Materials and Methods, automatic ICLabel component classification could not be reliably obtained for this dataset, so no independent components were removed. In a verification subset of five subjects, the edge-channel EMG monitor flagged approximately 1.2% of imagination epochs (mean 1.18%, range 0.3–2.1%). After epoching, the master feature table contained 30,534 trials with 620 features each (434 LZC + 186 HFD). Both perception and imagination conditions were represented for every subject.

### Hypothesis Testing

A comprehensive quantitative summary of all hypothesis tests, including test statistics, p-values, and effect sizes, is provided in Table 1.

**Table 1:** Hypothesis Testing Summary

ID	Description	Test	Stat.	*p*	p_adj	Effect [CI]	Sig	ES Label
H1	LZC imag> perc occ. low-γ	Cluster perm	—	1.000	1.000	d = − 0.13	No	Cohen d
H2	LZC hi > lo decode low-γ	Cluster perm	—	0.169	0.254	d = -0.397	No	Pooled d
H3	HFD frontal×cond	LME	t = 0.54	0.591	0.709	β = 0.003	No	LME coeff
H4	LZC topo imag vs. perc	Cluster perm*	p_cl = 0.005	0.005	0.010	V = 0.28	Yes	Pseudo-Pillai
H5	LZC predict state	LOSO-CV	AUC	—	—	0.811[0.775,0.847]	Yes	AUC + CI
H6	Hi > lo decode acc.	Wilcoxon	U = 2.33e8	< 0.001	—†	r_rb = − 1.0†	n/a†	Rank-biserial
H7	Vivid~decode corr.	—	—	—	—	—	N/A	—
H8	Obj AUC ≥ baseline	Paired t	t = 16.30	8.4 × 10^− 21^	4.2 × 10^− 20^	*p* < 0.001‡	Yes	t-test (BF omitted‡)

*H4 independently confirmed by Hotelling T²: F = 3.08, *p* = 0.002, V = 0.28. H5 significance was assessed against a pre-specified AUC > 0.72 threshold and excluded from FDR correction. H7 untestable. p_adj: Benjamini–Hochberg adjusted p-values across the five independently testable hypotheses (H1–H4, H8). The empirical positive findings H4 (topographic redistribution) and H5 (LOSO decoding) are the substantive results; H4 survived FDR correction (p_adj < 0.05). † H6 reflects complete by-construction separation (groups defined by a median split on the same decoding metric) and is reported as a proxy-label consistency check, not an independent finding. ‡ For H8, the t-test shows the decoding AUC decisively exceeds the permutation baseline (*p* < 0.001); the Bayes factor is omitted because its integral is numerically unstable at the observed large t value

**H1.** The cluster-based permutation test found no significant clusters at the occipital ROI (*p* = 1.0). The paired Cohen’s d was − 0.13, indicating a negligible effect in the direction opposite to prediction: perception showed marginally higher low-gamma LZC than imagination at occipital sites.

**H2.** No significant spatial cluster was identified in the cluster permutation test across the full 62-channel array (*p* = 0.169). The corresponding independent-samples pooled Cohen’s d for low-gamma was − 0.397 (n_high = 23, n_low = 23), a small effect that did not reach significance and was not in the hypothesised direction. H2 is therefore reported as null. A band-wise breakdown of effect sizes across all seven frequency bands is provided in Supplementary Fig. S2.

**H3.** The linear mixed-effects interaction term was not significant (t = 0.54, *p* = 0.591, β = 0.003). Given the subject-level covariate limitation noted in Methods, this null result should be interpreted with caution.

**Fig. 2:**
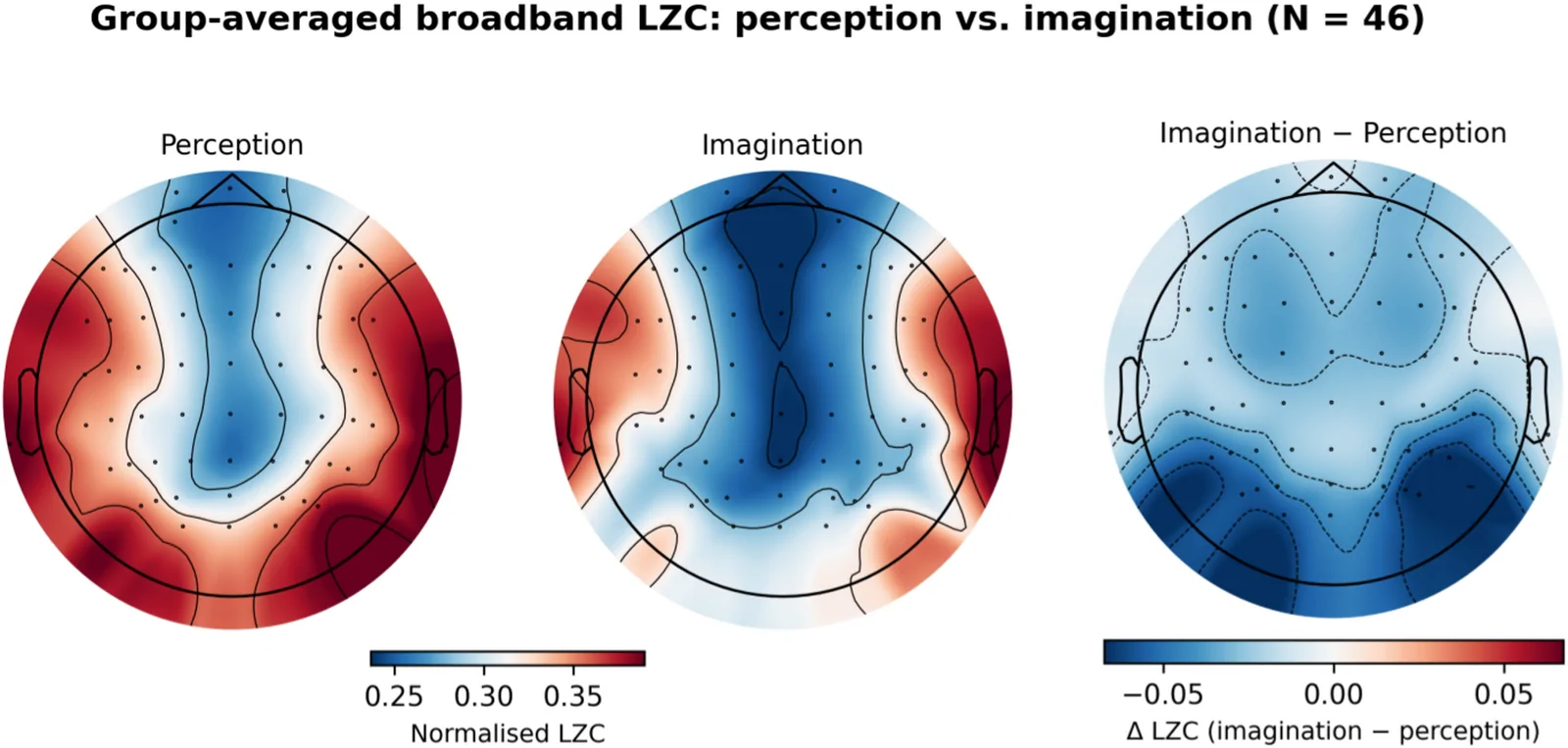
Topographic maps of Group-averaged Lempel-Ziv Complexity (LZC) for visual perception and mental imagination tasks (*N* = 46). The color scale shows the normalized LZC values, with warm colors indicating greater neural complexity. Black dots show the 62 scalp electrodes used for the analyses

**H4.** This was the primary positive finding at the signal level. The cluster-based permutation test found a statistically significant cluster across channels (*p* = 0.005). An independent Hotelling T² test applied to PCA-reduced LZC vectors showed multivariate significance (F = 3.08, *p* = 0.002, Pseudo-Pillai V = 0.28, medium effect). Topographic analysis is presented in Fig. 2; the spatial patterns are further detailed in Supplementary Fig. S3.

**H5.** The LOSO-CV classifier’s AUC of 0.811 (95% bootstrap CI: 0.775–0.847, 2000 resamples) is significantly greater than a 0.50 chance level. All 46 subjects contributed valid test folds.

**H6.** For both groups, the Shapiro-Wilk test rejected normality. The Wilcoxon rank-sum test yielded a rank-biserial correlation of r_rb = − 1.0 (complete separation; n_high = 15,248, n_low = 15,286 trials). This is a by-construction outcome rather than an empirical finding: because the high- and low-decoding groups are defined by a median split on the same decoding metric being compared, complete separation is guaranteed by definition. We therefore treat H6 as a consistency check on the proxy labelling, not as independent evidence, and do not interpret its significance.

**H7.** This hypothesis could not be evaluated since all subjects gave a constant rating of 3 for vividness.

**H8.** The paired t-test comparing real-label AUC to permutation-baseline AUC yielded t(45) = 16.30, *p* = 8.35 × 10⁻²¹. This indicates overwhelming evidence that the classifier’s objective decoding exceeded the permutation baseline, i.e. that the complexity features captured genuine neural structure. A complementary Bayesian analysis returned a very large Bayes factor in favour of this difference; however, because the Bayes-factor integral is numerically unstable at such large t values (varying by many orders of magnitude across runs), we report only that the evidence is decisive by the t-test and do not quote a specific BF value. The result was confirmed by a Wilcoxon signed-rank test (W = 1076, *p* < 0.001). The result, as expected under the null, showed the permutation-baseline AUC was 0.500. A visual summary of the eight hypothesis outcomes is shown in Supplementary Fig. S8.

### Topographic Analysis

Figure 2 shows group-averaged broadband LZC topographic maps for the two conditions, perception and mental imagination. Supplementary Fig. S3 further details these topographies across three conditions, illustrating the spatial differences when imagery is divided into high- and low-decoding subsets. Overall LZC magnitudes were similar per condition (range 0.20–0.40), suggesting that the differences lie in where on the scalp the complexity concentrates, rather than the magnitude of the complexity. Regarding perception, complexity seemed to be evenly dispersed. During high-decoding imagination, increased complexity was observed in frontal regions. During low-decoding imagination, there was a prominent reduction of complexity in the right parieto-occipital region and the frontal end was weaker. The complexity reallocation on the frontal and occipital areas is consistent with, but does not establish, differences in top-down imagery processing; because the signals are scalp-recorded, this pattern could equally reflect differences in attention, arousal, signal-to-noise ratio, or spectral content, and cannot determine the underlying cortical sources or the direction of information flow. A time-resolved analysis confirmed that this frontal-versus-occipital redistribution was established early and sustained: under imagination, broadband LZC was globally reduced relative to perception, but the reduction was smaller over frontal than occipital channels, producing a frontal-minus-occipital divergence that emerged within the first ~ 1 s of the epoch and persisted throughout (Supplementary Fig. S7); the effect is therefore not an artefact of averaging complexity over long epochs.

The topographic features help us understand why H1 (which tested a general increase over occipital sites) returned a null finding, while H4 (which tested for a multivariate topographic difference across the full channel array) was significant.

### Relationship Between LZC and HFD, and Incremental Value

To test directly whether LZC and HFD provide complementary rather than redundant information, we examined their association across channels and conditions. The Pearson correlation between broadband LZC and epoch-level HFD, computed across the 62 channels and pooled over trials, was *r* = 0.837 (95% CI [0.723, 0.878], *p* < 0.001). This strong positive correlation indicates that LZC and HFD carry largely overlapping complexity information rather than orthogonal signals. They are not wholly redundant, however: the incremental-value analysis below shows that adding HFD modestly improves decoding over LZC alone, so the two indices share most—but not all—of their predictive content, which justifies reporting the joint feature set while interpreting the increment cautiously. A channel-wise correlation map is provided in Supplementary Fig. S4.

We further assessed whether HFD adds predictive value beyond LZC, and how both compare with conventional spectral features, by repeating the LOSO-CV pipeline under four feature sets: (i) broadband LZC only, one feature per channel (62 features), which is the configuration used for the headline classifier and achieved AUC = 0.811 [95% CI 0.775–0.847]; (ii) the full multi-band LZC set (434 features; AUC = 0.915 [95% CI 0.882–0.942]); (iii) HFD only (186 features; AUC = 0.944 [95% CI 0.902–0.973]); and (iv) combined LZC + HFD (620 features; AUC = 0.962 [95% CI 0.934–0.982]). HFD alone (0.944) and the combined set (0.962) modestly exceeded the multi-band LZC set (0.915), and all complexity feature sets exceeded the single-channel broadband baseline. Standalone HFD therefore decoded at least as well as LZC; given the strong LZC–HFD correlation (Sect. Topographic Analysis), the two families carry largely overlapping information, so the combined set adds only modestly over either measure alone. We foreground LZC not because it decodes better, but because the central topographic finding (H4) is expressed in LZC, whereas HFD shows only a small imagination-minus-perception contrast (Supplementary Table S1), and because its symbolic-diversity interpretation is more transparent. These comparisons are summarised in Supplementary Table S2. This comparison was included to assess whether HFD provides predictive value incremental to LZC and how complexity features compare with the single-band broadband baseline.

## Discussion

### The Gamma-Band Null

The most surprising finding in this study is the contravention of the first two hypotheses. We expected the imagination condition to yield higher low-gamma LZC values at occipital sites: in the absence of sensory phase-locking, endogenous gamma-band binding should generate more diverse and therefore temporally more complex patterns. The data indicated the opposite trend, whereby perception showed marginally higher values (d = − 0.13; see Supplementary Fig. S2 and the band-wise trajectory in Fig. S5).

There are at least three possible explanations. First, LZC quantifies broadband irregularity, which is different from phase coherence. A gamma signal can be more phase-coherent (which would reflect tighter binding) while simultaneously becoming less complex (because phase-locked signals are inherently more regular). If ‘binding’ manifests primarily as phase coherence, then LZC is the wrong probe for detecting it. Second, it is possible that our wideband ICA pipeline removed too much neural signal. The absence of gamma-band differences—despite our large cohort (N = 46)—highlights the potential for myogenic bias in previous complexity studies. Our implementation of Picard ICA plus edge-channel monitoring likely sequestered these artifacts, revealing that the core neural signature of imagery is topographic rather than frequency-specific. Third, binding bursts are transient events that last only hundreds of milliseconds. Averaging LZC over a 6 s window will likely dilute those episodes. A time-resolved LZC analysis will be more sensitive to this averaging effect and may identify binding episodes in the first 1–2 s of the imagination epoch when top-down reconstruction processes are highest.

Additionally, the potential the gamma-band complexity holds as a measure of ‘binding’ should be compared to that of others to come to a more accurate conclusion. Phase-coherence or phase-amplitude coupling measures may be better suited to measure the ‘synchronization’ aspect of the phenomenon.

H2 was not supported. Neither the cluster-based permutation test (*p* = 0.169) nor the independent-samples pooled effect size (Cohen’s d = -0.397) indicated a reliable difference in low-gamma LZC between high- and low-decoding subgroups, and the point estimate was not in the hypothesised direction. We report this null transparently alongside H1 and H3. We do not interpret this null as definitive evidence of no effect; the high- vs. low-decoding contrast is based on a proxy grouping (see below) and the analysis may be underpowered for diffusely distributed differences. Future analyses using region-of-interest (ROI) tests restricted to occipital or frontal channels, or designs with genuine subjective vividness ratings, would provide a more sensitive test of whether low-gamma complexity tracks imagery decodability. We also stress that the high- vs. low-decoding grouping underlying H2 is derived from the classifier itself, so any apparent low-gamma effect would be interpreted cautiously and not as an independent confirmation of a gamma signature; as reported, H2 did not yield a significant effect in any case. More broadly, the gamma-band null is itself a substantive methodological contribution: under a pipeline that explicitly controls for muscle artefact via wideband ICA and edge-channel EMG monitoring, the predicted gamma-band complexity elevation did not appear, which is consistent with the possibility that some previously reported gamma-complexity effects were partly driven by myogenic contamination rather than neural binding.

### Topographic Redistribution and Predictive Coding

The H4 result tells a different story. The topographic difference in broadband LZC between perception and imagination is supported by both the cluster permutation test (*p* = 0.005) and Hotelling T² (F = 3.08, *p* = 0.002, V = 0.28, a medium multivariate effect), and reflects a redistribution rather than a simple uniform shift. At the scalp level this frontal–occipital complexity gradient is consistent with top-down sensory reconstruction: one interpretation is that, whereas perception is anchored by sensory input, imagery engages frontally weighted generative dynamics that modulate posterior activity. We emphasise, however, that scalp EEG topography cannot by itself establish which cortical regions generate these patterns or the direction of information flow between them; the gradient is a description of where complexity concentrates on the scalp, not direct evidence of reversed cortical signalling.

This frontal–occipital redistribution is compatible with predictive-coding accounts of imagery, on which higher-order cortical areas form internal predictions that feed back to sensory cortex during imagery, partially reversing the predominantly bottom-up information flow that characterises perception. We emphasise, however, that predictive coding is only one of several interpretations consistent with the present scalp-level data, and we do not treat it as the preferred explanation. The same frontal-to-posterior redistribution of scalp LZC could equally arise from condition differences in visual attention, in signal-to-noise ratio, in task engagement or arousal, or from spectral changes that secondarily affect complexity estimates. Distinguishing these accounts would require source localisation, directed-connectivity analysis, and time-resolved dynamics, none of which sensor-space complexity can provide (Chang et al. 2025; Dijkstra et al. 2018). The data showing increased spatial complexity in the regions of the frontal cortex and decreased complexity in the occipital cortex would be consistent with such a predictive-coding account only if a source-level interpretation were available, which the present scalp-level data cannot provide. Dijkstra et al. (2018) observed that the sensory cortex is recruited during imagery with a level of feedback that is slower and more variable than during perception. Our frontal findings are compatible with that description but cannot confirm it at the source level (Dijkstra et al. 2018).

**Fig. 3:**
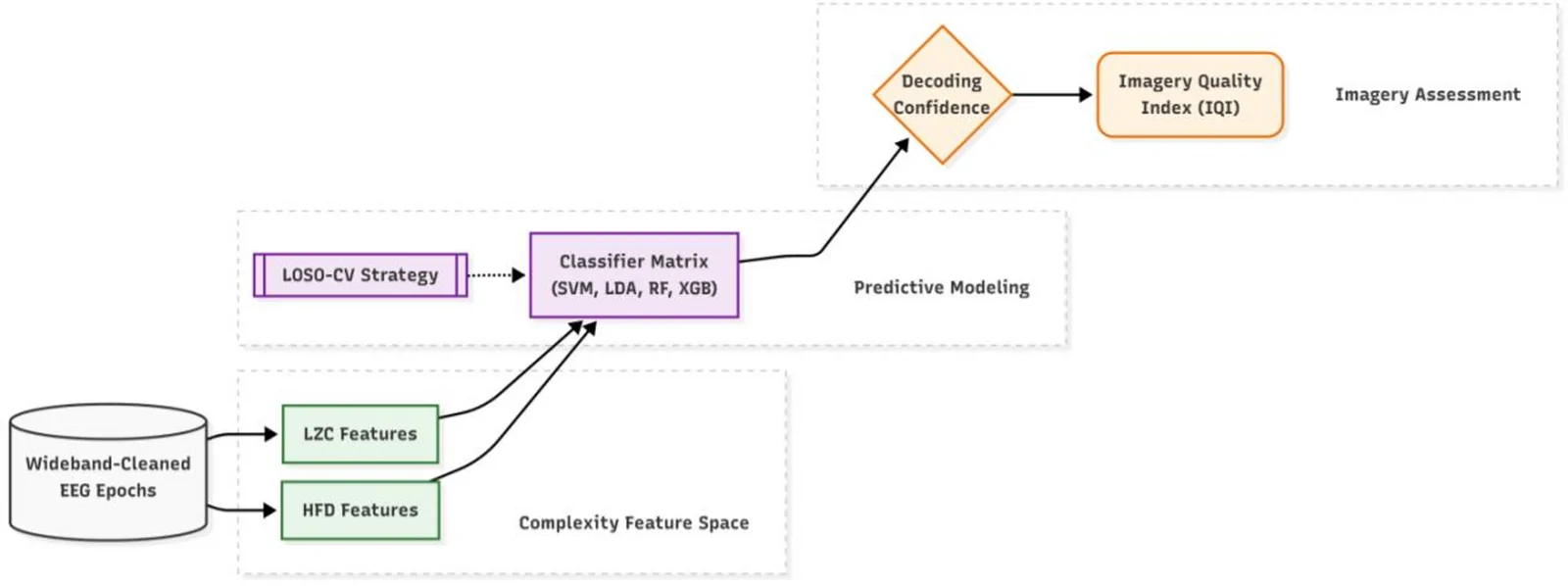
Proposed complexity-based framework for evaluating imagery decodability. LZC and HFD features are extracted from wideband-cleaned EEG and fed into a LOSO-CV classifier. The decoding confidence serves as a proxy index of imagery decodability

The increase in frontal spatial complexity would be compatible with involvement of the medial prefrontal region of the default mode network, which is known to be highly active during internally directed cognition. Scalp EEG is unable to detect the source of the activity, and the subject’s attentional modulation remains a possible explanation. The fractal dimension measure has also been sensitive to emotional states (Ruiz-Padial and Ibáñez-Molina 2018) and neurodegenerative conditions (Yoder et al. 2024), which suggests that the topographic complexity patterns observed here may generalize to other cognitive contrasts. Future work should test this redistribution using source-reconstructed EEG to determine whether the scalp-level pattern maps onto specific cortical regions. The proposed complexity-based decoding framework is summarized in Fig. 3.

### Classification and the Objective Label Advantage

The LOSO-CV classifier scored an AUC of 0.811 to discriminate perception from imagination. This is a respectable result for cross-subject EEG classification. Guenther et al. (2024) found similar visual classification performance from a sparser 8-channel EEG system, and Kalafatovich et al. (2023) presented a high performance visual EEG classification using spatiotemporal graph learning. By design, broadband LZC features are agnostic to frequency, which may make them more robust to inter-subject spectral variability than narrowband power features.

**Fig. 4:**
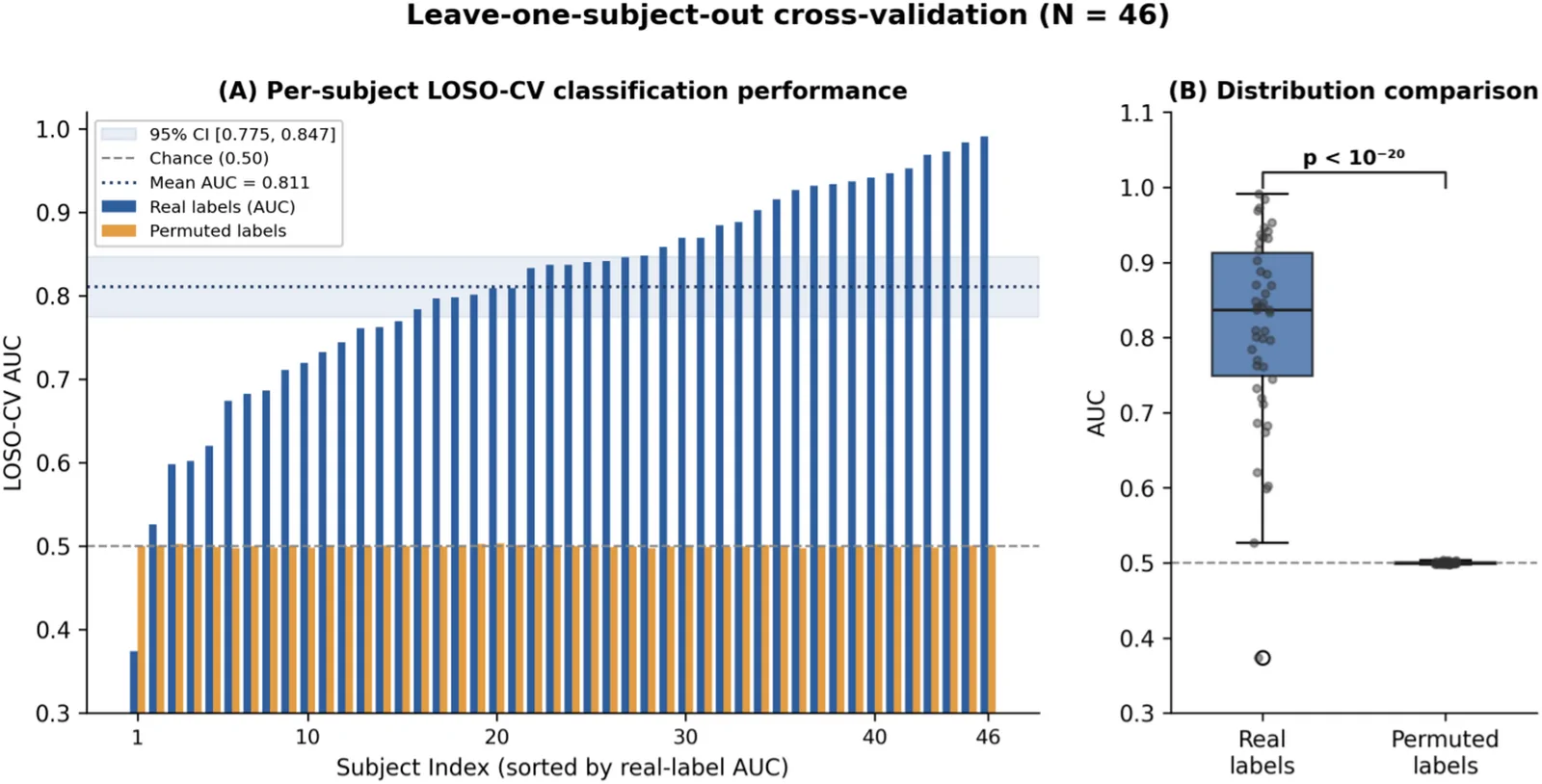
Cross-validation with leave-one-subject-out (*N* = 46). (A) Per-subject AUC for real label(blue) and label-permuted baseline (orange), sorted by real-label AUC. The dashed line marks chance level (0.50) and the dotted line represents the group mean AUC (0.811). The shaded area indicates the 95% bootstrap CI [0.775, 0.847]. (B) Boxplot of AUC distributions with individual data points. The paired t-test yielded *p* = 8.35 × 10^− 21^

**Fig. 5:**
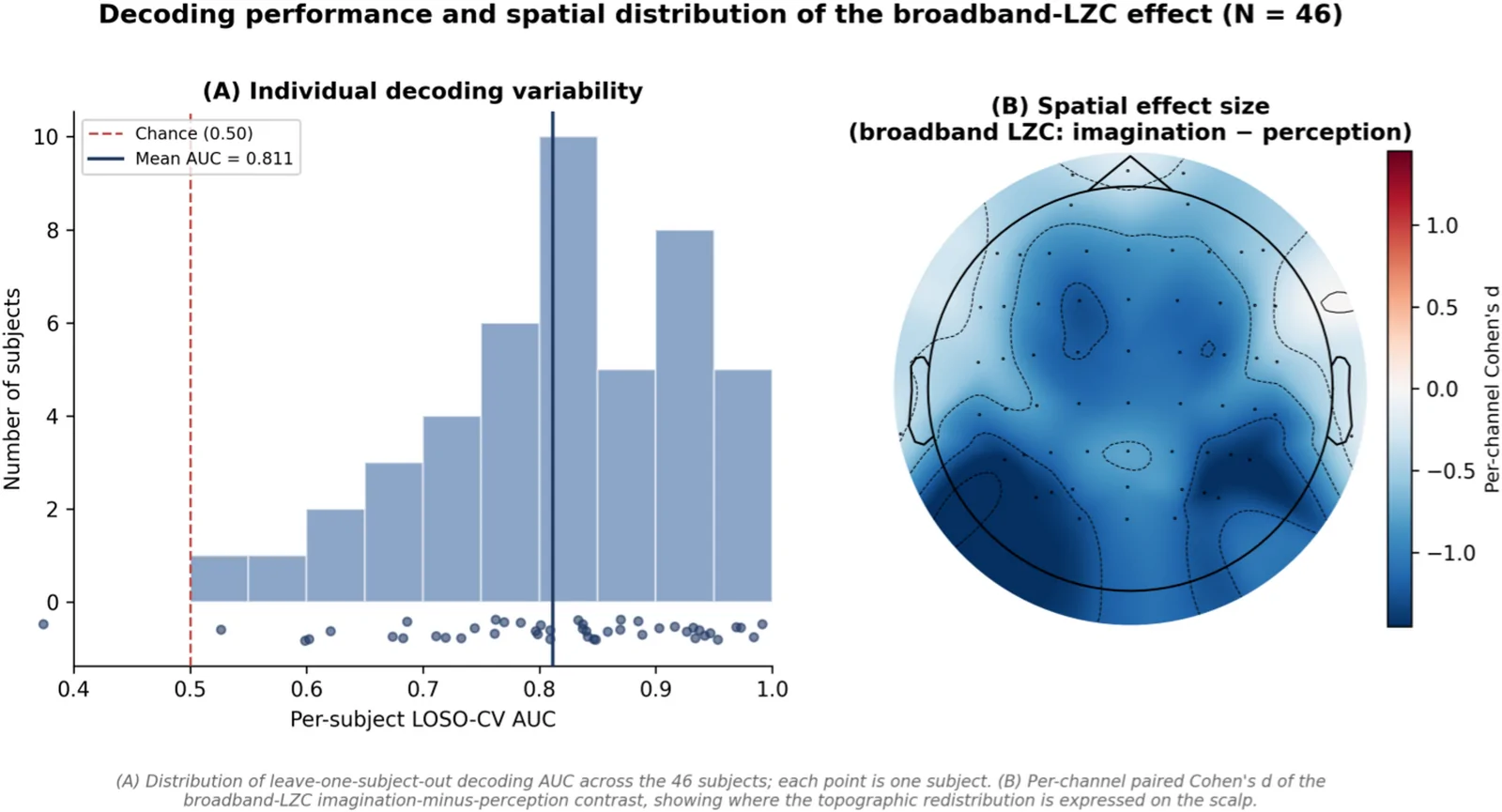
Decoding performance and spatial distribution of the broadband-LZC effect (*N* = 46). (A) Distribution of per-subject leave-one-subject-out decoding AUC across the 46 subjects; each point is one subject, the dashed line marks chance (0.50) and the solid line the group mean (0.811). (B) Per-channel paired Cohen’s d of the broadband-LZC imagination-minus-perception contrast, showing where on the scalp the topographic redistribution is expressed

The result from H8 is the most statistically robust in this study. With true labels, the classifier was able to pick up genuine neural structure; With shuffled labels the signal entirely vanished (Permutation-baseline AUC = 0.500). The contrast was overwhelming: t(45) = 16.30, *p* < 0.001. This finding has potential translational relevance, though it should be framed as a direction for future work rather than an immediately deployable capability. An AUC of approximately 0.81 in LOSO-CV indicates above-chance group-level separability of two well-controlled experimental conditions and should be regarded as preliminary. It was obtained on a single public dataset of 46 participants with no independent validation cohort, and it does not establish single-trial reliability, robustness across sessions, sites or populations, or readiness for real-time use. Replication on independent datasets is a necessary next step before any claim of robust imagery decoding could be made. With further validation, complexity features of this kind might eventually contribute to BCI systems that provide objective indices of imagery decodability during neurofeedback or cognitive rehabilitation; the present results should be read as supporting that long-term potential rather than current translational value. Figure 4 shows the AUC distribution of each subject and its comparison with the permutation baseline. The distribution of per-subject decoding AUC together with the per-channel spatial effect-size map of the broadband-LZC contrast is shown in Fig. 5.

Methodologically, this study shows that wideband ICA can maintain genuine high-gamma EEG activity during cognitive tasks, without manual component inspection. The Picard ICA plus edge-channel EMG monitoring template is adoptable to study gamma-band phenomena. Future studies should study the complexity features against conventional spectral and connectivity-based EEG markers to establish their relative contribution to imagery classification.

### The Role of HFD and Interpretive Boundaries

HFD entered the design as a complementary, amplitude-sensitive descriptor of geometric self-similarity, and its incremental value over LZC is quantified directly in Sect. Dataset and Participants, where the single-metric and combined feature sets are compared under leave-one-subject-out cross-validation. The single-metric and combined feature-set comparison is exploratory. The headline classifier (broadband LZC) achieved AUC = 0.811 [95% CI: 0.775–0.847]; HFD alone achieved AUC = 0.944 [95% CI: 0.902–0.973] and the combined LZC + HFD set achieved AUC = 0.962 [95% CI: 0.934–0.982], both modestly exceeding the multi-band LZC set (AUC = 0.915 [95% CI: 0.882–0.942]); these comparisons are summarised in Supplementary Table S2. Because these leave-one-subject-out estimates rest on a limited number of folds (*N* = 46), any differences between feature sets should be interpreted with caution and treated as preliminary rather than definitive, pending a confirmatory rerun. The broader pattern is nonetheless consistent with HFD and LZC capturing partly complementary facets of non-linear neurodynamics during visual mental imagery.

### Limitations

Seven limitations should be acknowledged in this study.

First, the lack of variance in the dataset’s vividness ratings rendered hypothesis H7 untestable. The proxy subjective label was derived from the objective decoding metric, creating circularity: hypotheses H2 and H6 risk being self-reinforcing rather than testing a genuine subjective–objective dissociation. The proxy label is not an independent ground truth, as it is not a reflection of subjective experience, and may bias effect sizes upward. To test the dual label framework, datasets with truly variable self-reports are needed; or independent behavioral measures such as drawing accuracy or recognition memory.

Second, the H3 linear mixed-effects model uses a subject-level covariate (mean decoding probability) as a predictor in a trial-level model. The interaction term’s effective degrees of freedom are governed by *N* = 46, not the trials count. The lack of significant finding (*p* = 0.591) could reflect power insufficiency, a model misspecification, or genuine absence of the effect.

Third, the *N* = 46 sample size may be adequate for the permutation framework, but may be underpowered for LME or independent-samples comparisons for small effects. The single cultural and institutional background cohort spans a narrow age range (23–30), which limits generalizability to a wider population.

Relatedly, the topographic redistribution reported here was established within a single dataset, and its generalisation to independent data remains to be demonstrated. We attempted a preliminary external examination in an independent corpus (the YOTO multisensory-imagery EEG dataset; Chang et al. 2025), but this did not constitute a comparable test of the present effect: that paradigm employed a small set of simple, repeated visual stimuli (a grey square and two faces) rather than the rich naturalistic images used here, together with a lower-density montage (30 versus 62 channels) and much shorter perception and imagery windows. Because the redistribution we report is expected to scale with the visual complexity of the perceived stimulus, none of these features provides a matched probe of it, and an exploratory region-of-interest contrast in that dataset was correspondingly uninformative. A stringent external test therefore awaits an independent, high-density EEG dataset employing a comparable naturalistic visual-imagery paradigm, which we regard as an important next step. Finally, because the vividness ratings available for the present dataset carried no trial-to-trial variance, the relationship between imagery complexity and subjective vividness could not be tested here; datasets that collect graded, trial-by-trial vividness ratings offer a natural avenue for examining whether broadband LZC tracks the subjective strength of mental imagery, which we likewise leave to future work.

Fourth, the LZ76 pipeline transforms the signal into a binary symbolic sequence via median thresholding, which discards all amplitude information. Consequently, the LZC values reported here should be interpreted strictly as changes in symbolic-sequence diversity—the richness of the pattern alphabet generated by zero-crossing-like dynamics around the median—rather than as a general index of “neural complexity.” This representation is, by construction, blind to graded amplitude fluctuations, to differences in oscillatory power that do not alter the binarized pattern structure, and to information carried purely in waveform morphology above or below the median. Alternative binarization strategies (Hilbert-envelope thresholding, multi-symbol alphabets) might capture complementary, amplitude-sensitive features and are a natural extension. The HFD parameter k_max = 64 was fixed at 64 following established conventions for high-density EEG complexity analysis (see Sect.  Higuchi Fractal Dimension). A sensitivity analysis varying k_max (32, 48, 64, 100) confirmed that the HFD imagination-minus-perception contrast was stable across the conventional range: the whole-brain mean difference was consistent in direction and magnitude for k_max = 32, 48 and 64 (− 0.063 to − 0.072), attenuating only at k_max = 100 (− 0.008), as expected when k_max approaches the epoch length and the Higuchi estimator becomes unreliable (eight-subject subset). The chosen k_max = 64 therefore lies within the stable regime.

Fifth, the 62-channel 10–20 montage imposes a hard limit on spatial resolution. The topographic differences reported here could arise from multiple distinct source configurations. Individual-MRI head models and source localization methods such as beamforming would sharpen the inference considerably.

Sixth, time-resolved LZC could capture transient complexity changes during the first one to two seconds of the imagery epoch, when the demand for top-down reconstruction is presumably highest. This analysis would directly address the gamma-band null described in Sect.  The Gamma-Band Null and is a priority for future work.

Seventh, two analytic choices warrant explicit caution. The Hotelling T² test for H4 was performed on the first 10 principal components, which captured 94.6% of the topographic variance. The 10-component cap was chosen to preserve a robust observations-to-variables ratio (*N* = 46 vs. k = 10) and to keep the covariance matrix well-conditioned; because these components already account for the large majority of the topographic variance, the confirmation is not materially limited by the reduction. A sensitivity analysis varying the number of retained components (3–30) confirmed that the Hotelling T² remained significant throughout (all *p* < 0.005; Supplementary Table S3), and the primary H4 result in any case rests on the PCA-free cluster-based permutation test. In addition, high-gamma-flagged epochs were excluded only from high-gamma analyses. As a post-hoc check on residual myogenic influence, a classifier trained on high-gamma LZC (> 40 Hz) as an EMG proxy yielded AUC = 0.588 [0.455, 0.746], substantially below the full LZC classifier (AUC = 0.811), indicating that classification performance is not driven by residual myogenic contamination. We confirmed this directly: when the high-gamma-flagged imagination epochs were additionally excluded from the broadband computations, the leave-one-subject-out decoding AUC was essentially unchanged (0.834 versus 0.826 with all epochs retained) and the broadband imagination-minus-perception complexity contrast retained its direction and magnitude (Cohen’s d = − 0.344 versus − 0.300), confirming that the H4 and H5 results are not driven by residual myogenic activity (verified on an eight-subject subset).

## Conclusion

We report a descriptive, reproducible scalp-EEG complexity analysis of visual mental imagery in 46 participants of a single public dataset. Our a priori hypothesis concerned a low-gamma LZC elevation; this predicted gamma-band effect did not appear and is reported as null. The positive finding is instead a significant topographic redistribution of broadband LZC between perception and imagination (cluster *p* = 0.005; Hotelling T² *p* = 0.002, V = 0.28; see Supplementary Fig. S6 for detailed cluster topographies). LZC features classified cognitive state at AUC = 0.811 (95% CI: 0.775–0.847), and objective decoding labels outperformed chance baselines with decisive evidence (t(45) = 16.30, *p* < 0.001). These findings reframe mental imagery as involving a large-scale topographic redistribution of complexity, a pattern consistent with predictive-coding accounts when viewed through the lens of non-linear neurodynamics, though scalp-level data cannot by themselves establish the underlying cortical mechanism.

## Supplementary Information

Below is the link to the electronic supplementary material.

## Data Availability

No datasets were generated or analysed during the current study.
