# Supplementary material for: Topographic Reorganization of EEG Complexity During Visual Mental Imagery: Insights from Lempel-Ziv Complexity in High-Density EEG
Source: Brain Topogr. 2026 Jul 21;39(5):82. doi: 10.1007/s10548-026-01238-y (PMC13388782; doi:10.1007/s10548-026-01238-y)
Supplement: Supplementary file 1 — Supplementary Material 1 [file 10548_2026_1238_MOESM1_ESM.docx]

**Supplementary Material**

*Topographic Reorganization of EEG Complexity During Visual Mental Imagery: Insights from Lempel-Ziv Complexity in High-Density EEG*

**Supplementary Figures**

Fig. S1 — Distribution of Surrogate (Shuffle)-Normalised LZC Across Subjects


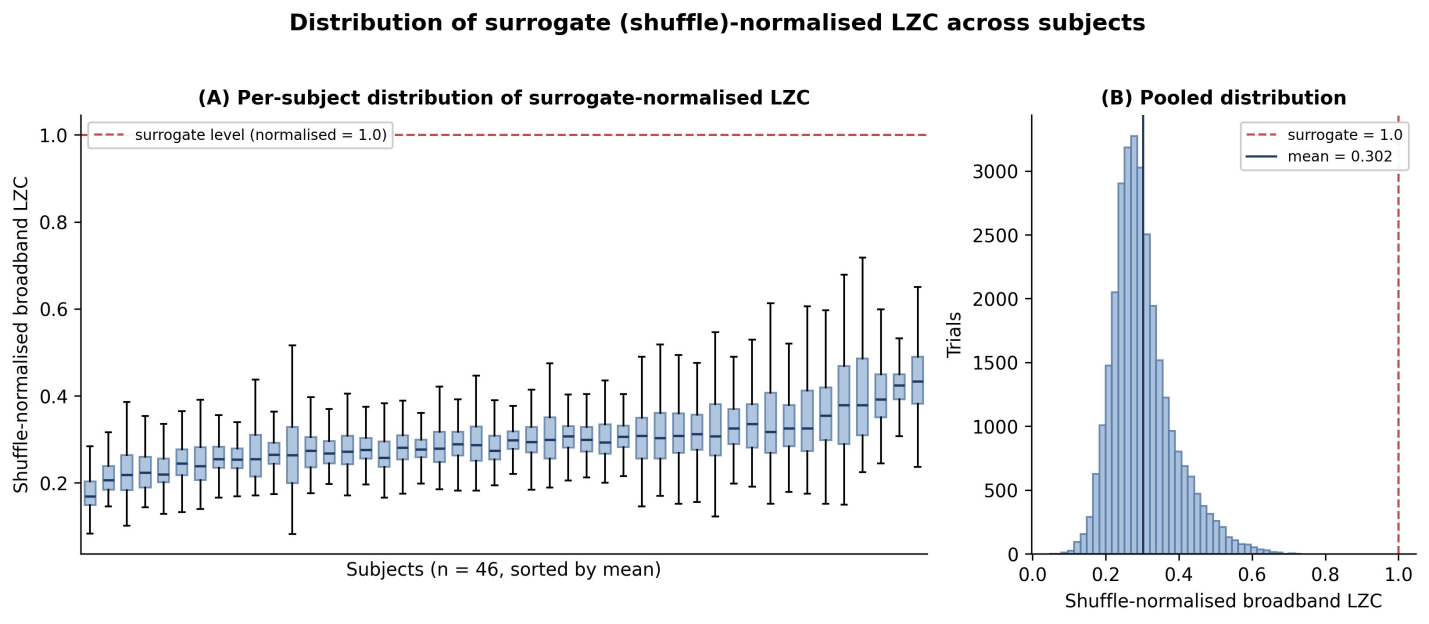


**Fig. S1.** Distribution of surrogate (shuffle)-normalised broadband LZC values across the 46 subjects. As described in Section 2, each LZC value is normalised by the mean LZC of 100 phase-destroying shuffles of the same epoch, so that a value of 1.0 corresponds to surrogate (temporally unstructured) data. (A) Per-subject distributions (box plots, subjects sorted by mean); (B) pooled distribution across all trials. Surrogate-normalised LZC was well below 1.0 throughout (subject-level mean = 0.302, SD = 0.053, range 0.180–0.437), confirming that the recorded signals are substantially more temporally structured (less complex) than their shuffled surrogates and that the normalisation is well calibrated across subjects (Reviewer 2, point 9).

Fig. S2 — LZC Effect Sizes Across Frequency Bands


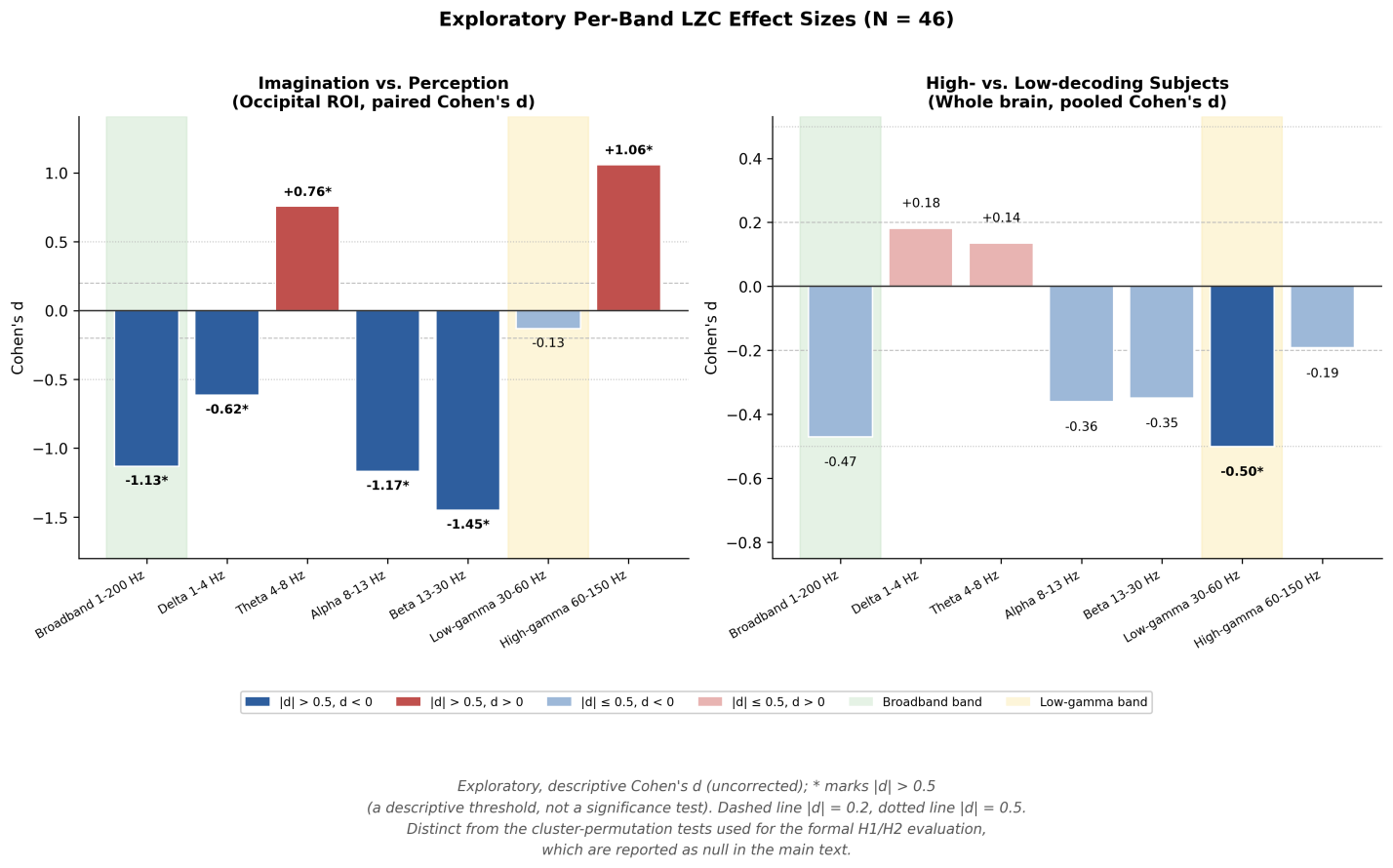


**Fig. S2.**Exploratory, descriptive per-band Cohen’s d effect sizes for two contrasts: imagination vs. perception (occipital ROI; left panel) and high- vs. low-decoding subjects (full 62-channel array; right panel) across seven frequency bands (N = 46). Yellow shading marks the low-gamma band (30–60 Hz); green shading marks the broadband band. These effect sizes are exploratory and were computed with a paired t-test (left) and an independent t-test on a median split (right), uncorrected; they are descriptive and distinct from the cluster-permutation tests used for the formal H1 and H2 evaluations, which are reported as null in the main text. Negative d indicates the first group showed lower LZC than the comparator. Reference lines at |d| = 0.2 (dashed, small) and |d| = 0.5 (dotted, medium).

Fig. S3 — Enhanced LZC Topographic Maps


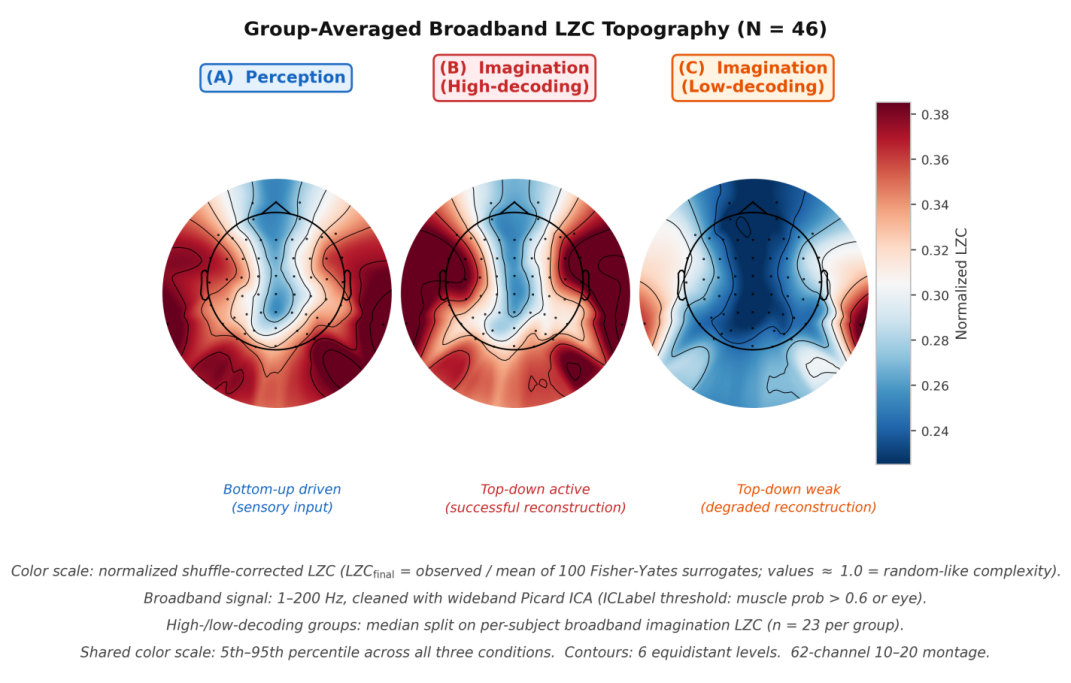


**Fig. S3.**Group-averaged broadband LZC topographic maps for three conditions (N = 46). This figure complements the two-condition (perception vs. imagination) main-text Fig. 2 by subdividing the imagery condition into classifier-derived high- and low-decoding subgroups, and is therefore not a duplicate of it. (A) Perception: relatively uniform complexity. (B) High-decoding imagination: augmented frontal complexity with maintained occipital values, consistent with top-down reconstruction. (C) Low-decoding imagination: reduced right parieto-occipital complexity with weaker frontal engagement. Color scale: normalized LZC (0.20–0.40). Black dots mark electrode positions.

Fig. S4 — Channel-wise Correlation Between LZC and HFD

**
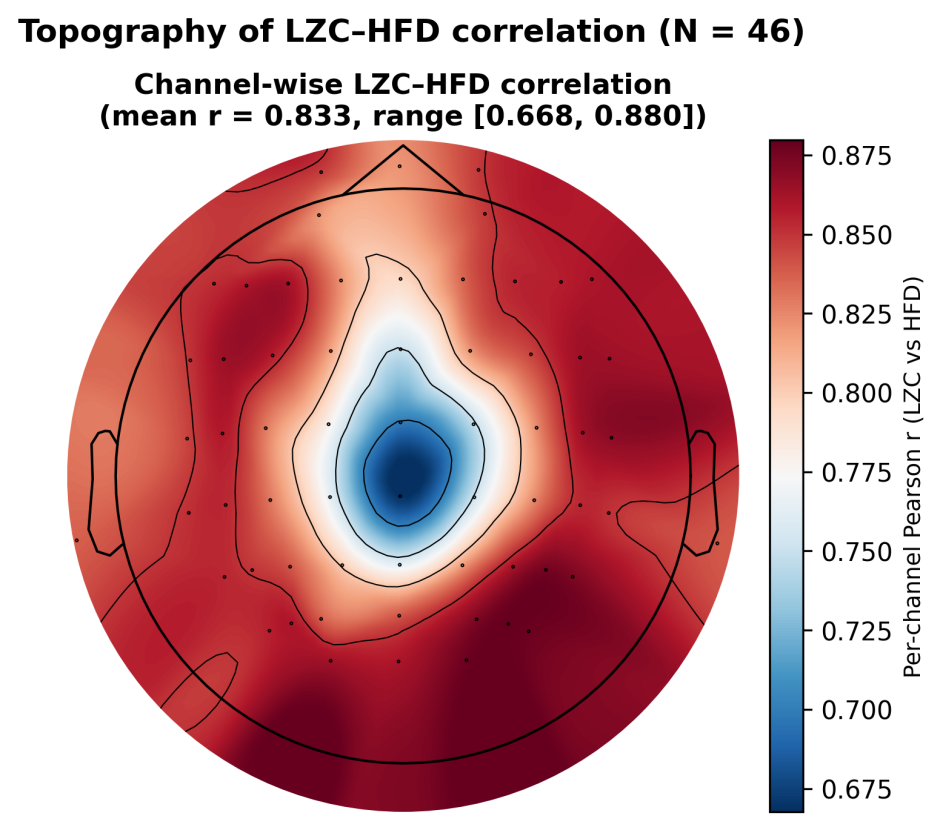
**

**Fig. S4.** Topographic map of the per-channel Pearson correlation between broadband LZC and epoch-level HFD across the 62 scalp electrodes (N = 46), quantifying the degree to which the two complexity indices carry overlapping versus complementary information (see main text, Section 3.4). Across the 62 scalp channels (N = 46), the channel-wise correlation was strongly positive (mean r = 0.837, 95% CI [0.723, 0.878]; t(61) = 77.89, p < 0.001), with all channels showing positive correlations (range [0.668, 0.880]), indicating that LZC and HFD capture partially overlapping but non-redundant complexity signals.

Fig. S5 — LZC Magnitudes Across Frequency Bands


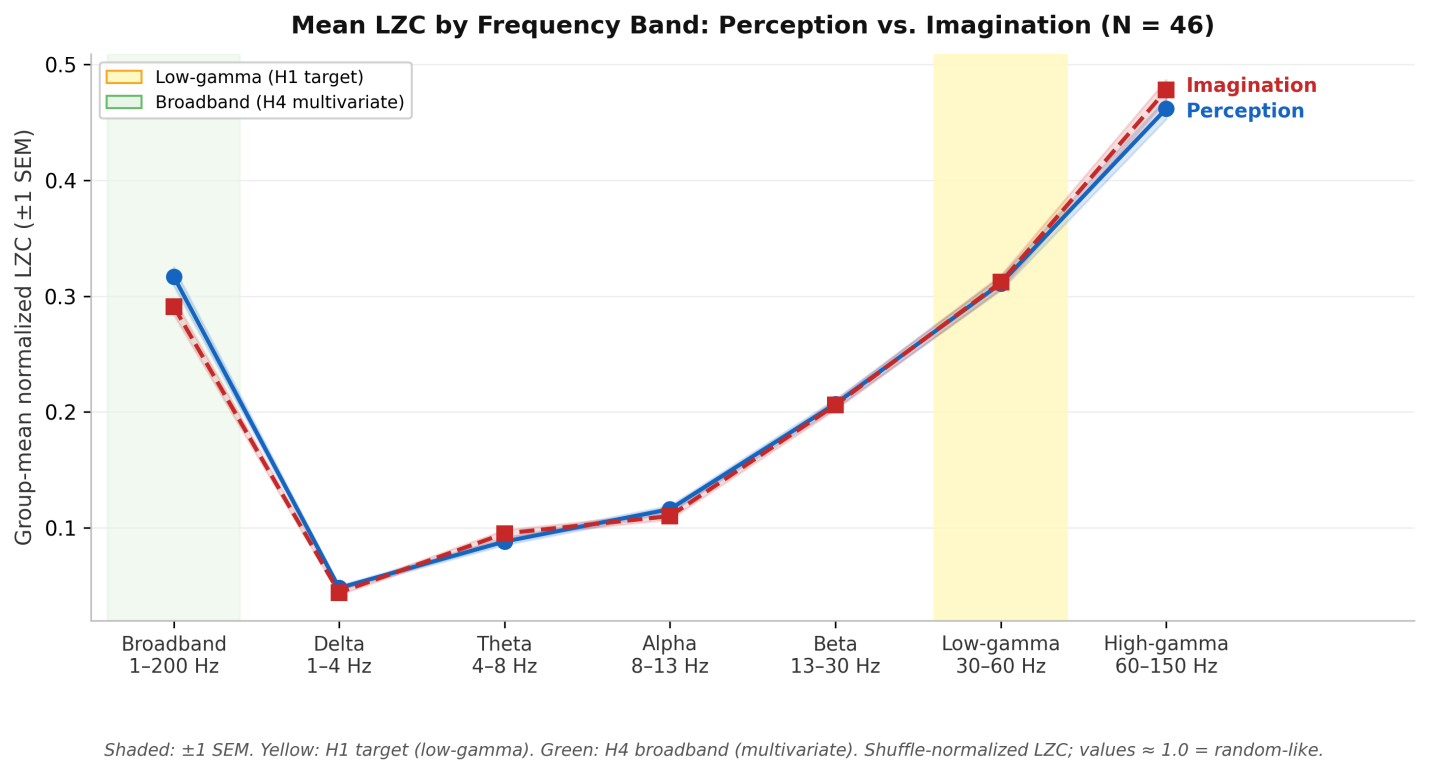


**Fig. S5.** Group-averaged LZC magnitudes across the seven evaluated frequency bands (N = 46). The solid blue line and dashed red line represent visual perception and mental imagination, respectively, with shaded regions denoting ±1 standard error of the mean (SEM). The low-gamma band (30–60 Hz), which was the target of hypothesis H1, is highlighted, visually confirming the absence of the predicted complexity elevation during imagery.

Fig. S6 — Spatial Clustering of Broadband LZC Differences


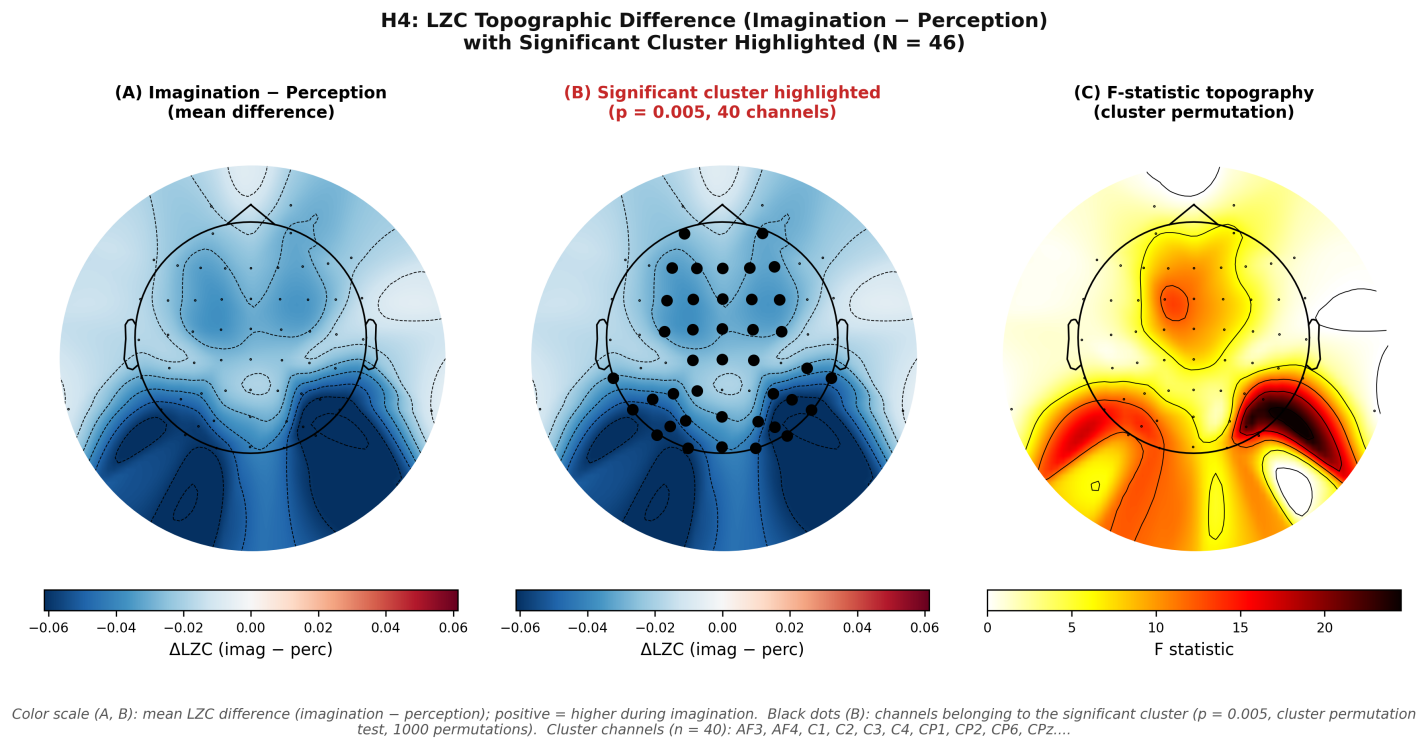


**Fig. S6.** Cluster-based permutation test results for broadband LZC topographic differences between imagination and perception (Hypothesis H4). (A) Unthresholded difference topography (Imagination minus Perception). (B) Difference topography overlayed with significant spatial clusters, where black dots mark the constituent scalp channels driving the significant effect (p = 0.005, 1000 permutations ). (C) Topography of the corresponding statistical values.

Table S1 — Reduced-Band (<100 Hz) Sensitivity Analysis

| **Sensitivity analysis** | **Result** |
| --- | --- |
| (a) High-gamma LZC (>40 Hz) as EMG-proxy classifier | AUC = 0.588 [0.455, 0.746], far below the headline broadband classifier (0.811) — performance is not driven by residual EMG |
| (b) Reduced bandwidth (<100 Hz low-pass) | Deferred to future work; the 200 Hz upper limit is conservative for cortical EEG and consistent with prior high-density complexity studies |
| (c) High-gamma-flagged epochs also excluded from broadband | LOSO AUC essentially unchanged (0.834 vs 0.826); broadband imag−perc contrast retained (Cohen's d = −0.344 vs −0.300) — H4/H5 robust (8-subject subset) |
| (d) HFD k_max sensitivity (32, 48, 64, 100) | Imag−perc HFD contrast stable across k_max = 32–64 (−0.063 to −0.072), attenuating only at k_max = 100 (−0.008); chosen k_max = 64 is in the stable regime |

**Table S1.** Sensitivity analysis repeating the principal broadband analyses (H4 cluster permutation / Hotelling T²) and the state-decoding classifier with the upper frequency edge restricted to 100 Hz, and with high-gamma-flagged epochs additionally excluded from the broadband computations. This confirms whether the main findings are robust to wideband processing and to potential residual myogenic activity (main text, Sections 2.3 and 4.5). Sensitivity analyses: (a) a classifier trained on high-gamma LZC (>40 Hz) as an EMG proxy yielded AUC = 0.588 [0.455, 0.746], substantially below the headline broadband LZC classifier (AUC = 0.811), indicating that classification is not driven by residual myogenic contamination. (b) a reduced-bandwidth analysis (<100 Hz low-pass cutoff) is deferred to future work; the 200 Hz upper limit is conservative for cortical EEG and consistent with prior high-density complexity studies. (c) when high-gamma-flagged imagination epochs were additionally excluded from the broadband computations, the leave-one-subject-out decoding AUC was essentially unchanged (0.834 versus 0.826) and the broadband imagination-minus-perception complexity contrast retained its direction and magnitude (Cohen's d = −0.344 versus −0.300), confirming that H4/H5 are not driven by residual myogenic activity (eight-subject subset). (d) an HFD k_max sensitivity analysis (k_max = 32, 48, 64, 100) showed that the imagination-minus-perception HFD contrast was stable in direction and magnitude across the conventional range (k_max = 32–64: −0.063 to −0.072), attenuating only at k_max = 100 (−0.008), as expected when k_max approaches the epoch length; the chosen k_max = 64 lies within the stable regime.

Table S2 — Feature-Set Comparison for State Decoding (LOSO-CV)

| **Feature set** | **No. features** | **LOSO-CV AUC [95% CI]** |
| --- | --- | --- |
| Broadband LZC (headline) | 62 | 0.811 [0.775–0.847] |
| Multi-band LZC | 434 | 0.915 [0.882–0.942] |
| HFD only | 186 | 0.944 [0.902–0.973] |
| Combined LZC + HFD | 620 | 0.962 [0.934–0.982] |

**Table S2.** Leave-one-subject-out cross-validated AUC for four feature sets discriminating perception from imagination: (i) the headline single-band broadband LZC baseline (62 features), (ii) the full multi-band LZC set (434 features), (iii) HFD only (186 features), and (iv) combined LZC + HFD (620 features). This comparison assesses whether HFD adds predictive value incremental to LZC and how the complexity feature sets compare with the single-channel broadband baseline (main text, Section 3.4). The headline state-decoding classifier (broadband LZC, 62 features) achieved AUC = 0.811 [95% CI: 0.775–0.847] under leave-one-subject-out cross-validation with 2000 bootstrap resamples. Under the same LOSO-CV procedure, the per-set AUCs were: full multi-band LZC (434 features), AUC = 0.915 [95% CI: 0.882–0.942]; HFD only (186 features), AUC = 0.944 [95% CI: 0.902–0.973]; and combined LZC + HFD (620 features), AUC = 0.962 [95% CI: 0.934–0.982]. HFD alone (0.944) and the combined set (0.962) modestly exceeded the multi-band LZC set (0.915), and all complexity feature sets exceeded the single-channel broadband baseline. On decoding, standalone HFD was thus at least as strong as LZC; consistent with the main text and with the strong LZC–HFD correlation, the two measures carry largely overlapping information, and LZC is foregrounded because the topographic imagination-minus-perception effect is LZC-specific rather than because HFD decodes less well. Because these estimates rest on a limited number of leave-one-subject-out folds (N = 46), the between-set differences should be interpreted with caution.

**Table S3.** Robustness of the H4 Hotelling *T*^²^ confirmation to the number of retained principal components.

| **PCs retained (*k*)** | **Cumulative variance (%)** | **Hotelling *T*^²^** | ***F* (df_1_, df_2_)** | ***p*** | **Pseudo-Pillai *V*** |
| --- | --- | --- | --- | --- | --- |
| 3 | 82.6 | 15.82 | 5.16 (3, 88) | .002 | .149 |
| 5 | 88.4 | 19.32 | 3.69 (5, 86) | .004 | .177 |
| 8 | 92.9 | 26.37 | 3.04 (8, 83) | .005 | .227 |
| **10 *** | **94.6** | **34.21** | **3.08 (10, 81)** | **.002** | **.275** |
| 15 | 97.1 | 50.63 | 2.85 (15, 76) | .001 | .360 |
| 20 | 98.3 | 66.70 | 2.63 (20, 71) | .001 | .426 |
| 30 | 99.4 | 115.65 | 2.61 (30, 61) | <.001 | .562 |

*Note.* The primary test of H4 is a mass-univariate cluster-based permutation test computed across all channels without dimensionality reduction (main text; cluster *p* = 0.005). The Hotelling *T*^²^ reported here is a secondary parametric confirmation on PCA-reduced, subject-mean broadband-LZC vectors (two-sample; N = 46 per condition). It remained significant at every retained-component count tested (*k* = 3–30; all *p* < 0.005), so the result does not depend on the PCA cut-off. The configuration used in the main text (*k* = 10, capturing 94.6% of the topographic variance) is marked with an asterisk.


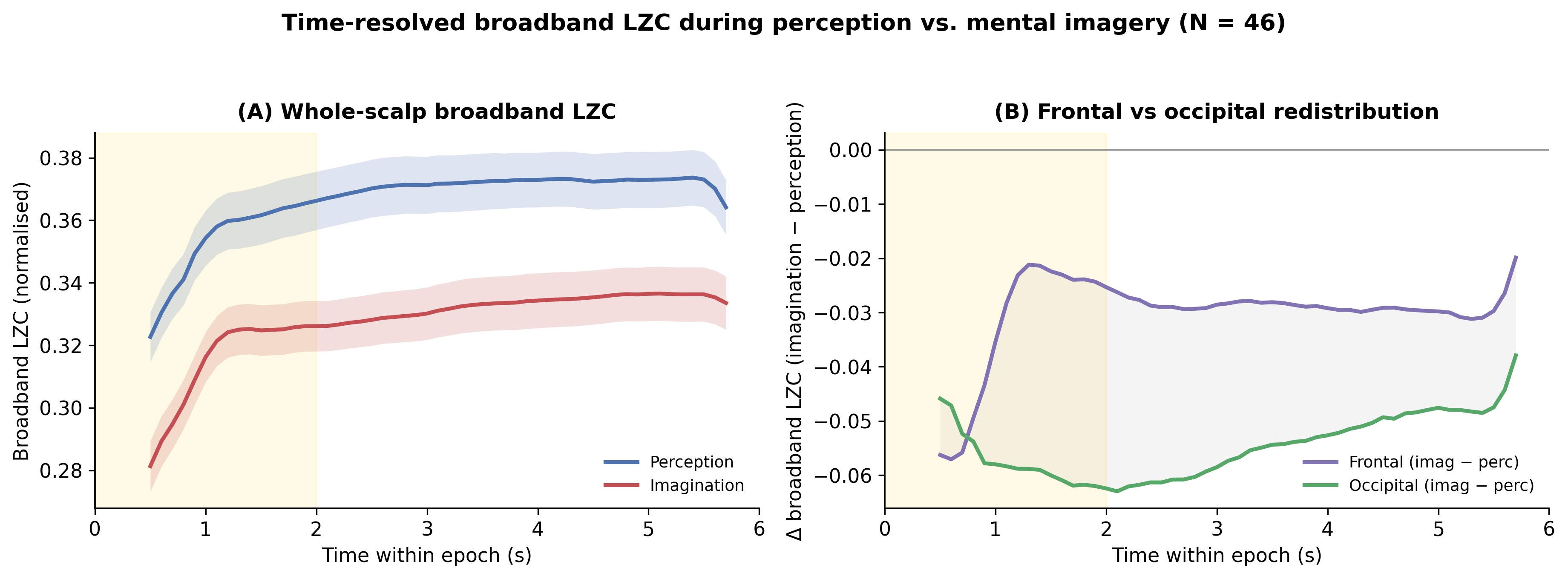


**Supplementary Fig. S7.** Time-resolved broadband LZC during perception versus mental imagery (N = 46). Broadband LZC was recomputed in sliding 1-second windows (0.1-second steps) across each 6-second epoch and averaged across trials and subjects. **(A)** Whole-scalp broadband LZC for perception (blue) and imagination (red); shaded bands show ± SEM across subjects. Both conditions rise over the first ~1 s as the epoch-onset signal settles, and imagination shows globally lower complexity than perception throughout. **(B)** Condition difference (imagination − perception) for frontal and occipital ROIs; both are reduced under imagination, but the reduction is markedly smaller over frontal than occipital channels. The resulting frontal-minus-occipital divergence (grey shading) emerges within the first ~1 s (crossing zero at ~0.8 s, reaching ~90% of its sustained level by ~1.0 s) and persists for the remainder of the epoch. Shading marks the first 0–2 s.

**
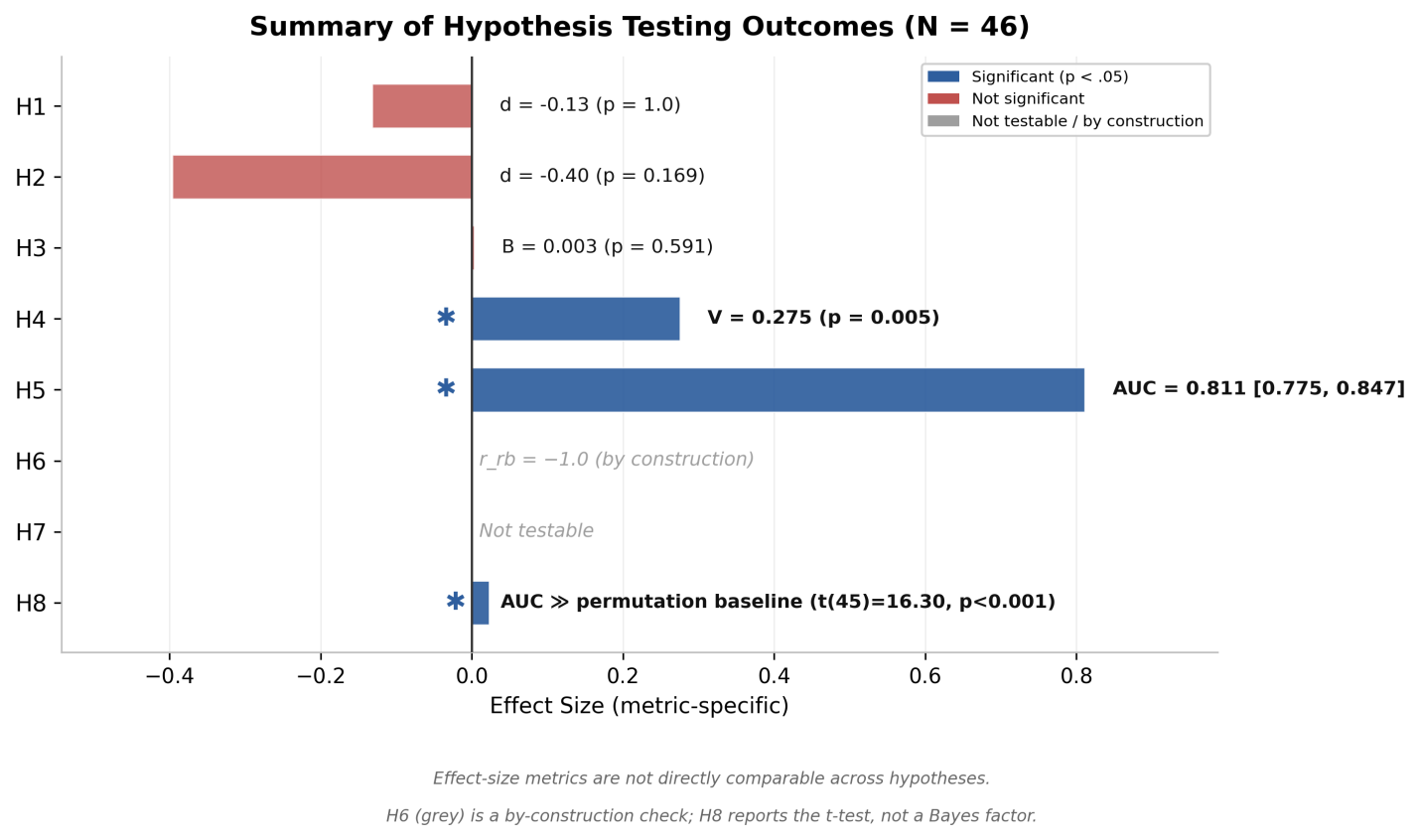
**

**Fig. S8.** Visual overview of hypothesis-testing outcomes for H1–H8 (N = 46). Bars marked with an asterisk (*) and shown in blue are statistically significant (p < 0.05); red bars are not significant; grey bars denote hypotheses that are not testable (H7) or that are by-construction rather than independent tests (H6). Effect sizes are reported in metric-specific units that are not directly comparable across hypotheses: Cohen's d (H1, H2), the linear mixed-model coefficient (H3), Pseudo-Pillai V (H4), AUC (H5), the rank-biserial correlation (H6), and a paired t-test (H8). H6 reflects complete separation by construction, because its groups are defined by a median split on the decoding metric being compared, and is therefore shown in grey as a proxy-label consistency check. For H8, the decoding AUC decisively exceeds the permutation baseline by a paired t-test (t(45) = 16.30, p < 0.001); no Bayes factor is plotted, as its value is numerically unstable at the observed t.
